# Expanding the phenotype of *PRPS1* syndromes in females: neuropathy, hearing loss and retinopathy

**DOI:** 10.1186/s13023-014-0190-9

**Published:** 2014-12-10

**Authors:** Berta Almoguera, Sijie He, Marta Corton, Patricia Fernandez-San Jose, Fiona Blanco-Kelly, Maria Isabel López-Molina, Blanca García-Sandoval, Javier del Val, Yiran Guo, Lifeng Tian, Xuanzhu Liu, Liping Guan, Rosa J Torres, Juan G Puig, Hakon Hakonarson, Xun Xu, Brendan Keating, Carmen Ayuso

**Affiliations:** Center for Applied Genomics, The Children’s Hospital of Philadelphia, Philadelphia, PA 19104 USA; College of Life Sciences, University of Chinese Academy of Sciences, Beijing, 100049 China; BGI-Shenzhen, Shenzhen, 518083 China; Department of Genetics and Genomics, IIS-Fundación Jiménez Díaz University Hospital (IISFJD, UAM), 28040 Madrid, Spain; Center for Biomedical Network Research on Rare Diseases (CIBERER), ISCIII, Madrid, Spain; Department of Ophthalmology, Fundación Jiménez Díaz, 28040 Madrid, Spain; Department of Neurology, Fundación Jiménez Díaz, 28040 Madrid, Spain; Department of Biochemistry, La Paz University Hospital IdiPaz, Madrid, 28046 Spain; Department of Internal Medicine, Metabolic-Vascular Unit, La Paz University Hospital IdiPaz, Madrid, 28046 Spain; The Guangdong Enterprise Key Laboratory of Human Disease Genomics, Shenzhen, China

**Keywords:** *PRPS1*, Retinitis pigmentosa, Non-random X-chromosome inactivation, Phosphoribosyl pyrophosphate synthetase deficiency, Neuropathy

## Abstract

**Background:**

Phosphoribosyl pyrophosphate synthetase (PRS) I deficiency is a rare medical condition caused by missense mutations in *PRPS1* that lead to three different phenotypes: Arts Syndrome (MIM 301835), X-linked Charcot-Marie-Tooth (CMTX5, MIM 311070) or X-linked non-syndromic sensorineural deafness (DFN2, MIM 304500). All three are X-linked recessively inherited and males affected display variable degree of central and peripheral neuropathy. We applied whole exome sequencing to a three-generation family with optic atrophy followed by retinitis pigmentosa (RP) in all three cases, and ataxia, progressive peripheral neuropathy and hearing loss with variable presentation.

**Methods:**

Whole exome sequencing was performed in two affecteds and one unaffected member of the family. Sanger sequencing was used to validate and segregate the 12 candidate mutations in the family and to confirm the absence of the novel variant in *PRPS1* in 191 controls. The pathogenic role of the novel mutation in *PRPS1* was assessed *in silico* and confirmed by enzymatic determination of PRS activity, mRNA expression and sequencing, and X-chromosome inactivation.

**Results:**

A novel missense mutation was identified in *PRPS1* in the affected females. Age of onset, presentation and severity of the phenotype are highly variable in the family: both the proband and her mother have neurological and ophthalmological symptoms, whereas the phenotype of the affected sister is milder and currently confined to the eye. Moreover, only the proband displayed a complete lack of expression of the wild type allele in leukocytes that seems to correlate with the degree of PRS deficiency and the severity of the phenotype. Interestingly, optic atrophy and RP are the only common manifestations to all three females and the only phenotype correlating with the degree of enzyme deficiency.

**Conclusions:**

These results are in line with recent evidence of the existence of intermediate phenotypes in PRS-I deficiency syndromes and demonstrate that females can exhibit a disease phenotype as severe and complex as their male counterparts.

**Electronic supplementary material:**

The online version of this article (doi:10.1186/s13023-014-0190-9) contains supplementary material, which is available to authorized users.

## Background

Phosphoribosyl pyrophosphate synthetase 1 (PRS-I, [MIM 311850]) is an ubiquitous enzyme with an essential role in nucleotide metabolism: it catalyzes the synthesis of phosphoribosyl pyrophosphate (PRPP), the substrate for the synthesis of purine, pyridine, and pyrimidine nucleotides [1]. PRS-I has two highly homologous isoforms, PRS-II (MIM 311860) widely expressed as PRS-I, and PRS-III (MIM 611566) whose expression is restricted to testes. The three isoforms are products of separate but highly conserved genes: *PRPS1* (Xq22.3), *PRPS2* (Xp22.2)*,* and *PRPS1L1* (7p21.1), respectively [2]. Missense mutations in *PRPS1* are rare and may result in increase or decrease in PRS-I activity. PRS-I deficiency is an extremely rare condition that can lead to three different disorders: Arts syndrome (MIM 301835), being the most extreme form of enzyme deficiency; Rosenberg-Chutorian syndrome or X-linked Charcot-Marie Tooth 5 (CMTX5, MIM 311070), which represents a less severe phenotype; and X-linked non-syndromic sensorineural deafness (DFN2, MIM 304500), as the mildest form of deficiency. Only twelve families have been described so far with PRS-I deficiency syndromes [3-7] [I. del Castillo, personal communication]. Hearing loss is the only common feature between the three disorders and the only symptom observed in DFN2. Arts syndrome and CMTX5 share additional neurological anomalies such as ataxia, hypotonia, and optic atrophy [4,8,9]. Arts syndrome is also characterized by delayed motor development, mild to moderate intellectual disability and frequent recurrent infections that typically result in early death [3,10]. PRS-I superactivity (MIM 300661) is more frequent than the deficiency and is characterized by hyperuricemia and gout and it can be accompanied by other neurological symptoms such as sensorineural deafness, hypotonia, mental retardation, and also by recurrent infections [11,12]. All four disorders are inherited in a recessive X-linked manner so only males are affected. Obligate female carriers, however, may occasionally display milder symptoms such as hearing loss in CMTX5 [8,9], hearing impairment, ataxia, hypotonia or hyperreflexia in Arts syndrome [3], or hyperuricemia in PRS-I superactivity [13].

Using whole exome sequencing (WES), we identified a novel loss-of-function mutation in *PRPS1* leading to enzyme deficiency in three females with optic atrophy (OA), retinitis pigmentosa (RP), ataxia, peripheral neuropathy and hearing loss with variable presentation.

## Methods

### Subjects

**Figure 1:**
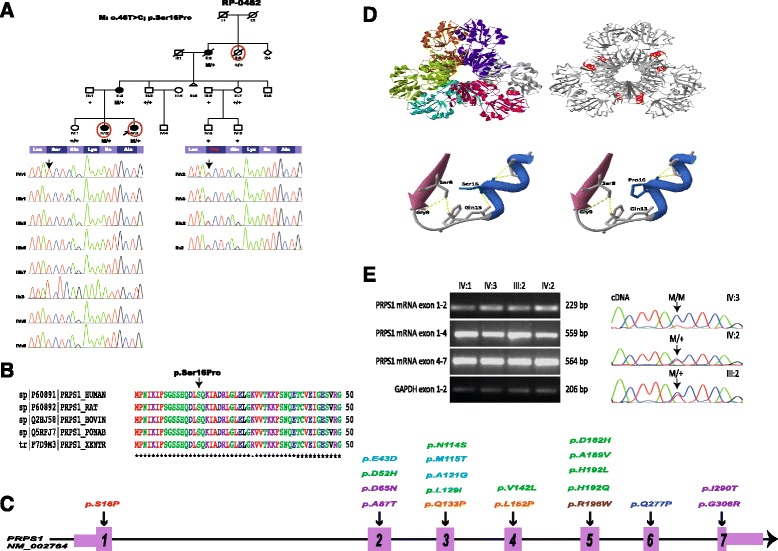
***PSPS1*** **is mutated in females with syndromic retinitis pigmentosa from a three-generation family. A.** Pedigree of family RP-0482 and validation by Sanger sequencing of p.Ser16Pro demonstrating the correct segregation in the family. All affected individuals (II:2, III:2; IV:2 and IV:3) are heterozygous for the variant. Red circles indicate the individuals analysed by WES. Although II:2 and II:3 died during the course of the study, DNA samples and informed consents were obtained before deceasing, allowing us to include those subjects in the segregation analysis. **B.** Multiple sequence alignment of *PRPS1* across species using ClustalW [14] confirms that p.Ser16 is identical from human to zebrafish. **C.** Schematic representation of *PRPS1* with the location of the novel heterozygous mutation p.Ser16Pro in exon 1 (in red) and previously known mutations in Arts syndrome (in orange), PRS-I Superactivity (in green), Charcot-Marie-Tooth disease-5 (in blue), X-linked nonsyndromic sensorineural deafness (in purple), and also the recently reported mutation associated with retinal dystrophy (in brown). Exons are indicated by rectangles that are wider for the coding regions. Nucleotide numbering reflects cDNA in the reference sequence NM_002764. **D.** Model of PRS-I with p.Ser16Pro based on the crystal structure of human PRS-I (PDB: 2H06) and close-up of the mutation showing the loss of a hydrogen bound with residue Gln13. **E.** RT-PCR and sequencing analysis of p.Ser16Pro in mRNA. RNA was derived from peripheral blood lymphocytes of the three patients (IV:2, IV:3 and III:2) and an unaffected control (IV:1). Normally spliced amplicons of exons 1–2, 1–4 and 4–7 of *PRPS1* comprising 229, 559 and 564 nucleotides, respectively, were found in all cases. Amplification of *GAPDH* mRNA analysis was used as positive control. Sanger sequencing of RT-PCR products evidences the absence of the wild-type allele in the cDNA of the proband (IV:3).

A three-generation Spanish family (RP-0482) consisting of four affected females (Figure 1A) was recruited and evaluated by the Fundacion Jimenez Diaz Hospital (Madrid, Spain). The four affecteds, six unaffecteds and two unrelated members of the family participated in the genetic study. All four affected females, II:2, III:2, IV:2 and IV:3, displayed typical or sectorial RP and various degrees of neurological symptoms. II: 2 and II:3 died during the course of the study but we obtained their DNA samples and informed consent before allowing us to include them in the study. Additionally, 191 unrelated Spanish individuals with no history of retinal dystrophy and randomly selected from blood donors voluntarily participated as controls. Informed consent was obtained from all individuals involved, all procedures were reviewed and approved by the Ethics Committee of the Hospital and adhered to the tenets of the Declaration of Helsinki.

### Whole exome sequencing and variant analysis

Genomic DNA was captured by Agilent SureSelect Human All Exon kit version 2 covering 46 MB of coding region (Agilent Technologies, Santa Clara, CA, USA), and sequenced on HiSeq 2000 instruments (Illumina, San Diego, CA, USA). Raw reads were mapped to the human reference genome (UCSC hg19), using the Burrows–Wheeler alignment tool [15]. Genome Analysis Tool Kit version 1.4 [16] was used for variant calling. ANNOVAR [17] was used for variant functional annotation and prediction and conservation scores from SIFT [18], Polyphen2 [19], LRT [20], MutationTaster [21], PhyloP [22] and GERP++ [23] were retrieved from the Database for Nonsynonymous SNPs’ Functional Predictions [24] for every potential nonsynonymous single nucleotide variant (SNVs).

Variant filtering was performed under the assumption of dominant inheritance. Only nonsynonymous, splice site disrupting, and frameshift heterozygous variants segregating in the family with a minor allele frequency ≤0.5% in a control cohort of more than 8000 individuals (1000 Genomes Project, [25] (April, 2012 release); 6503 exomes from NHLBI GO Exome Sequencing Project [26], and 669 in-house whole-exomes) were considered. Further gene prioritization was performed combining data on minor allele frequency, sequence conservation, potential deleterious effect and biological and clinical relevance according to gene function, gene expression, and the existence of mutation reports in databases such as The Human Gene Mutation Database [27], The Retinal Information Network (RetNet) [28] or Online Mendelian Inheritance in Man (OMIM) [29].

### Sanger sequencing

Sanger sequencing was used for the segregation of all variants resulting from the filtering analysis in family RP-0482 and to confirm the absence of variant p.Ser16Pro in *PRPS1* in 191 Spanish controls (primers available in Additional file 1). PCR products were enzymatically purified with ExoSAP-it (USB, Affymetrix), sequenced on both strands using Big Dye Terminator Cycle Sequencing Kit v3.1 (Applied Biosystems) and resolved on an automated sequencer (ABI 3130xl Genetic Analyzer, Applied Biosystems).

### *In silico* analyses of p.Ser16Pro pathogenicity

The evolutionary conservation of p.Ser16Pro was assessed by multiple sequence alignment of *PRPS1* across species using ClustalW2 [14]. The impact of the mutation in the tridimensional structure of the protein was assessed with a model of PRS-I with p.Ser16Pro based on the crystal structure of the human protein (PDB: 2H06) using Swiss Model [30,31] and Swiss PDB viewer [32]. ESEFinder [33,34] was used to determine whether p.Ser16Pro could alter the normal splicing of the mRNA.

### Determination of PRS activity in erythrocytes

PRS enzymatic activity was determined in erythrocytes from III:2, III:3, IV:1, IV:2, and IV:3, according to the method previously described by Torres et al. [35]. Interval used as reference was 70–126 nmol/h/mg hemoglobin.

### *PRPS1* expression analysis

*PRPS1* expression analysis was performed in RNA samples using blood lymphocytes from III:2, IV:1, IV:2, and IV:3. Total RNA was reversely transcribed to cDNA with ImProm-II™ Reverse Transcription System (Promega) using random primers. RT-PCR experiments were performed using *PRPS1* exonic primers pairs spanning exons 1–2, 1–4, and 4–7 (primers available in the Additional file 1). Primers for the housekeeping *GAPDH* gene were used as internal control. PCR fragments were subjected to electrophoresis in a gel searching for abnormally spliced amplicons and further sequenced.

### X-chromosome inactivation

X chromosome inactivation assay was performed in sodium bisulfite-treated genomic DNA from peripheral blood and saliva (EpiTect Bisulfite Kit, Quiagen) by a methylation specific PCR of the first exon of the human androgen receptor *locus* with fluorochrome-coupled primers. Heterozygosity of the human androgen receptor region in the target samples was previously checked. Two different pairs of primers were used to detect the methylated and unmethylated alleles and PCR fragments were analyzed in an ABI PRISM 3100 Genetic Analyzer (Applied Biosystems). X chromosome inactivation skewing was reported as percentage of the predominant allele and was considered skewed when the predominant allele exceeded 74%, non-skewed between 50% and 65% and undetermined between 66% and 74% [36].

## Results

### Familial history of RP-0482

**Figure 2:**
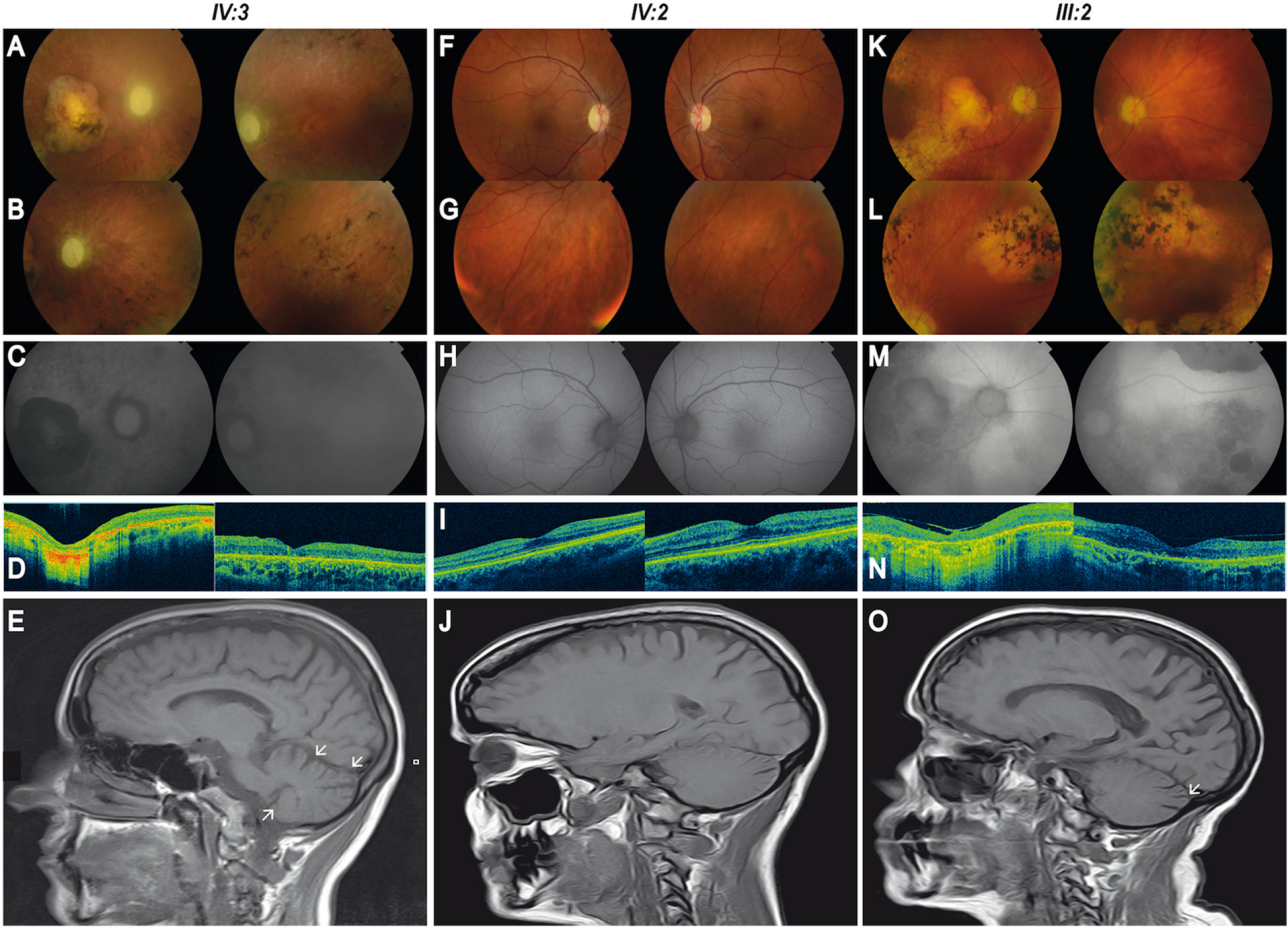
**Retinal and head MRI imaging of patients carrying the p.Ser16Pro variant in** ***PRPS1*** **. A-E.** Retinal imaging of both eyes (BE) of the proband IV:3 at age 35. **(A-B)** Fundus photographies show pale and atrophic papilla, narrowed vessels, diffuse hypopigmentation, bone spicules, and retinal pigment epithelium (RPE) atrophy in the mid-periphery of BE, and a well-defined atrophic macular lesion in the right eye (RE). **(C)** Autofluorescence reveals hypofluorescence areas corresponding with pigmented spicules and atrophic lesions. **(D)** Optical coherence tomography (OCT) confirms the generalized atrophy in all retinal layers in both perifoveal and foveal regions in RE. Well-conserved retinal architecture was found in the left eye (LE) and the atrophy is restricted to RPE and external layers. **(E)** Head MRI scan of the proband IV:3 demonstrates a moderate cerebellar atrophy (white arrows). **F-J.** Retinal imaging of BE of IV:2 at age 37. **(F-G)** Fundus photographies show a slight pallor in the papilla, slight arteriolar attenuation mainly around the optic nerve, a normal macula and an incipient RPE atrophy. **(H)** Normal autofluorescence. **(I)** OCTs display atrophy of photoreceptors layer in the perifoveal region and normal foveal thickness. **(J)** Head MRI scan of individual IV:2 does not evidence any sign of cerebellar atrophy. **K-O.** Retinal imaging of BE of III:2 at age 70. **(K-L)** Fundus photographies evidence pale and atrophic papilla, substantially narrowed vessels, sparse pigmentation, patches of chorioretinal atrophy in the mid-periphery of BE, and a well-defined atrophic macular lesion in RE and RPE alteration in LE. **(M)** Autofluorescence imaging shows alternating areas of hypo and hyperfluorescence. **(N)** OCT shows subfoveal atrophy lesion with loss of retinal architecture, an epiretinal membrane in RE and defects in external layer in LE. **(O)** Head MRI scan of individual III:2 exhibits a mild cerebellar atrophy (white arrow).

Data from ophthalmological, neurological and otological examinations of patients III:2, IV:2, and IV:3 were available from a period of more than 15 years and the phenotype is described in detail in the Additional file 1. Patient II:2 also displayed sectorial RP and ataxic traits, but the progression of the clinical phenotype is not available. Figure 2 shows the retinal and MRI images of the three affected females. Age of onset, presentation and severity of the phenotype are highly variable in the family, as summarized in Table 1. Both the index patient and her mother have various degrees of ataxia, peripheral neuropathy and hearing loss beyond the ophthalmological symptoms, whereas the phenotype of the affected sister is currently confined to the eye and milder than those of III:2 and IV:3 (Additional files 1 and 2)

**Table 1:** **Summary of findings in family RP-0482 and comparison with other PRS-I deficiency disorders (adapted from de Brouwer et al.** [11]**)**

	**IV:3**	**IV:2**	**III:2**	**Arts syndrome**	**CMTX5**	**DFN2**
Date of birth	1978	1976	1943			
Symptoms (age at diagnosis)						
Ophthalmological						
Retinitis Pigmentosa	+ (14y)	+ (23y)	+ (47y)	-	-	-
Night blindness	++ (4y)	+ (16y)	+	-	-	-
Visual field constriction	++	+	+	-	-	-
Visual acuity loss	++ (4y)	-	++	-	-	-
ERG alteration	++	+/−	++	-	-	-
Pigmentary changes at fundus	+	+/−	+	-	-	-
Macular atrophy	++	+	+	-	-	-
Optic atrophy	+ (5y)	+ (16y)	+ (47y)	+	+ (~10y)	+
Nystagmus	+ (Congenital)	-	-	-	-	-
Cataracts	+	-	+	-	-	-
Hyperopia	+	+	+/−	-	-	-
Hearing impairment	++ (21y)	-	+ (50y)	++	+	+
Neurological	++ (34y)	-	+ (55y)			
Mild developement delay	+/−	-	-	+	-	-
Hypotonia	+	-	-	+	+	-
Delayed motor development	-	-	-	+	-	-
Peripheral neuropathy	++	-	+	+	+	-
Pes cavus	+	-	-	NR	NR	NR
Loss of deep tendom reflexes	-	-	-	-	+	-
Cerebellar Atrophy	++	-	+			
Ataxia	+	-	+	+	+	-
Essential tremor	+	+	+			
Symptoms in carrier females	+++	+	++	Isolated and milder	Hearing loss	None
PRS activity erythrocytes (nmol/h/mg Hb) (Reference: 70–126)	Erythrocytes: 10	Erythrocytes: 41	Erythrocytes: 65	Erythrocytes: No activity	Fibroblasts: Decreased	Erythrocytes and fibroblasts: Decreased
				Fibroblasts 13-fold decrease		
				EBV-LCLs: normal		
Structural effect of mutation	Whole protein structure?			ATP site and allosteric sites I and II	ATP site and allosteric site I	Local structure

The number of “+” indicate the severity of the manifestation. NR = Not reported and EBV-LCLs = Epstein-Barr virus–transformed lymphoblastoid cell lines.

### Identification of a novel missense mutation in *PRSP1* by WES

DNA samples from individuals III:3, IV:2, and IV:3 were subjected to WES (Figure 1A). A total of 10.77 GB data on target genomic regions was generated for the three samples, with a mean coverage of target region of 78.23X. An average of 48,306 SNVs and 8,218 insertion/deletion (indels) were called for the three exomes, but for further filtering only coding variants were considered, thus reducing the number to an average of 18,722 SNVs and 741 indels per sample. Variant filtering under the assumption of a dominant inheritance yielded 141 variants in 126 genes as potential candidates (Additional file 3) with no known cause of retinal degeneration amongst them (RetNet, [28] accessed June, 2013). Further filtering of the 141 variants, left 12 novel variants, conserved across species according to the values from PhyloP and GERP++, predicted to be pathogenic at least by two of the systems evaluated and with expression in the retina, so they were selected for validation and segregation in the family (Additional file 1). Only the novel missense change in exon 1 of *PRPS1*, c.46 T > C; p.Ser16Pro (NM_001204402), completely segregated in all four affecteds and eight unaffected members of family RP-0482 (Figure 1A) and was absent in controls (258 X chromosomes total). Both GERP++ and PhyloP values estimated a high degree of conservation of serine at position 16 of PRS-I across species (Additional file 3), and was confirmed by multiple sequence alignment (Figure 1B), being the first missense mutation identified in this gene from the first α-helix to the fourth β-strand (aminoacids 1 to 43, Figure 1C). All *in silico* programs evaluated except for Polyphen2 predicted p.Ser16Pro to be damaging (Additional file 3). The tridimesional model of PRS-I with p.Ser16Pro indicated that Ser16 is located in the first α-helix of the protein in the N-terminal domain (Figure 1D). The replacement of serine with proline leads to the loss of a hydrogen bond with Gln13 likely breaking the tightly packed α-helix.

### p.Ser16Pro leads to PRS deficiency in females

To further assess the functional effect of the mutation in vitro, PRS enzymatic activity was determined in erythrocytes from three affected females (III:2, IV:2, and IV:3), where different levels of enzyme deficiency were evidenced (Table 1), and two unaffecteds (III:3 and IV:1) with PRS-I within the normal range.

### Lack of expression of *PRPS1* p.Ser16 in the index patient

The *in silico* analysis using ESEFinder predicted this mutation to alter the recognition pattern of splicing RNA proteins compared to the wild type sequence. To confirm this, *PRPS1* expression analysis was performed in RNA samples from blood lymphocytes from three affecteds and one unaffected (IV:1). RT-PCR analysis yielded no differences on the expression of *PRPS1* transcripts between carriers of p.Ser16Pro and the non-carrier (Figure 1E), and no additional splicing transcripts were found. Notably, further sequencing of mRNA transcripts evidenced the mutation in homozygosis in the index patient (Figure 1E).

### Non-random patterns of X-chromosome inactivation in the index patient

Non-random patterns of X-chromosome inactivation were assessed in the three affecteds (III:2, IV:2 and IV:3) and one unaffected individual (IV:1). Only in IV:3, there was a significantly skewed inactivation (82%) of the paternal allele, which explains the lack of expression of the wild type allele observed in the mRNA (Figure 1E). Individuals IV:1, IV:2 and III:2, had 50%, 61% and 64% X-chromosome inactivation, respectively, so they were considered non-skewed.

## Discussion

Here we report a novel mutation in *PRPS1* leading to PRS-I deficiency in three-females from a family with a phenotype consisting of OA followed by RP in all cases, plus neurological features overlapping CMTX5 and Arts syndrome with variable presentation in the proband (IV:3) and her mother (III:2).

Despite being already described that carrier females of *PRPS1* mutations can exhibit PRS-I deficiency and a disease phenotype [3,7-9], to date, no female has been reported to display such a complex and severe phenotype as observed in this family, specially in the proband. This patient showed a phenotype with features of Arts syndrome and CMTX5, with optic atrophy as the first symptom, followed by RP and neurological manifestations, such as hearing loss, intellectual disability and peripheral neuropathy (Table 1). The findings from this patient are consistent with the increasing evidence of intermediate phenotypes in PRS-I syndromes, recently described by several authors [6,7,12,37]. In particular, Synofzik and colleagues recently described a male with an intermediate phenotype between CMTX5 and Arts syndrome and a carrier female affected with DFN2 due to X-chromosome inactivation skewing [7]. The authors observed a correlation between the enzymatic residual activity, the degree of X chromosome inactivation skewing and the phenotype in the female and postulated that the location of the mutation and the residual enzymatic activity would be the main determinants of the phenotypic manifestations in males and females, respectively [7]. The three affected females from our family exhibited different levels of PRS deficiency in erythrocytes, ranging from a severe decrease in the activity in IV:3 to an almost normal activity in III:2. However, non-random patterns of X-chromosome inactivation were exclusively observed for the index patient (IV:3), who was found to only express the mutant allele in lymphocytes. In this patient, there was an apparent correlation between the X-chromosome inactivation in leukocytes, the lack of expression of the wild type allele in lymphocytes, the degree of PRS deficiency in erythrocytes, and the severity of the phenotype. X chromosome inactivation in subjects III:2 and IV:2 was not skewed and the degree of PRS-I deficiency only correlated with the age of onset of ophthalmological symptoms but not with the presentation or severity of the phenotype. This lack of correlation is not surprising given that X-inactivation, whether random or biased, occurs in a tissue-specific manner and what is observed in a particular tissue or cell type, such as leukocytes may not be representative of the status of central and peripheral nervous systems [7,38], which are primarily affected in PRS-I deficiency syndromes. Given the variable expression of the disease in the family it is very possible that either X-inactivation is skewed in those target systems or that the expression of PRS-II is compensating PRS enzymatic function as previously suggested, and so phosphoribosyl pyrophosphate synthesis would not be critically affected [11].

A relationship between the level of PRS-I disruption and the different syndromic manifestations has been suggested [11], with the most severe syndromes caused by mutations predicted to impact allosteric and active sites and those responsible for milder phenotypes disrupting the structure locally [11]. Very recently, Al-Maawali and colleagues expanded the *PRPS1* phenotype to retinal dystrophy and diabetes insipidus in a family with two males affected with Leber’s congenital amaurosis along with other manifestations, and no carrier female affected [37]. The mutation responsible for the phenotype was located in exon 5 of the gene (p.Arg196Trp), close to the ATP binding site of PRS-I. Residue Ser16 is located in the N-terminal domain of the protein, in a region not previously found mutated (Figure 1C) and far from the allosteric and active sites, both associated with Arts syndrome, CMTX5, and the recent retinal dystrophy phenotype, all features harbored by this family. That demonstrates once again the mutation-specific nature of PRS-I phenotypes [7,11]. Nevertheless, the existence of a putative defective splicing isoform, as predicted by *in silico* analysis cannot be ruled out, as it would be degraded by nonsense-mediated decay and thus, would not be detected by RT-PCR. In addition, other genes or epigenomic factors could be contributing to the complexity and severity of the phenotypes of PRS-I syndromes. Finally, it is worth noting that we cannot rule out either a dominant inheritance pattern for this particular mutation with a lethal effect in hemizygotes due to the lack of affected males in the family.

## Conclusion

These results support previous findings evidencing the existence of intermediate phenotypes in PRS-I deficiency syndromes and demonstrate that female carriers of *PRPS1* mutations can be as severely affected as their male counterparts and therefore, these syndromes may need also to be considered in females even in the absence of affected males in the family.

## Additional files

Additional file 1:
**Clinical data of family RP-0482 and primers used in the study.**
Additional file 2: Figure S1. Multifocal ERG and Optical coherence tomography (OCT) findings suggested a sectorial affectation of perifoveal photoreceptors. A. Right eye multifocal ERG (mfERG) records in patient IV:2 demonstrate a reduced function of photoreceptors of perifoveal region with preserved function of fovea. The ring analysis (see schematic) goes from the centre to the periphery. Quantitative results of mfERG analyses are displayed in table format. B. OCT macular cube 512×128 scan show macular thickness in the right eye from patient IV:2. ILM: inner limiting membrane; RPE: retinal pigment epithelium. Top left: fundus image with scan cube overlay. Top right: macular thickness significance map. The central innermost 1-mm-diameter circle represents the central subfield; inner superior, inner nasal, inner inferior, and inner temporal areas bounded by the 3-mm-diameter circle form the inner macula; outer superior, outer nasal, outer inferior, and outer temporal areas bounded by the 6-mm-diameter circle form the outer macula. Retinal thickness values from ILM to RPE are compared to the normative data. Middle and bottom left: cross-sectional OCT scans. Middle right: 3days surface maps: the ILM-RPE, displaying the retinal thickness in three dimensions. Bottom right: central subfield thickness, overall average macular thickness, and overall macular volume compared to normative data are displayed in table format. Reduction in the retinal thickness in the perifoveal region with a normal foveal thickness is also evidenced. Additional file 3: Table S1 and S2. Genetic variants obtained (single nucleotide variants –SNV-, Table S1, and insertion deletion variants –indels-, Table S2) after the first variant filtering analysis. **Table S3.** Variants yielded by the second filtering of the 141 variants based on the novelty, conservation across species according to the values from PhyloP and GERP++, predicted pathogenicity at least by two of the systems evaluated and expression in the retina. 1KGP: Minor allele frequency in the 1000 Genome Project, ESP: Minor allele frequency in the NHLBI GO Exome Sequencing Project. For PhyloP prediction, C: Conserved, NC: Not conserved. For pathogenicity prediction systems, B: benign, D: deleterious (LRT), or damaging (SIFT) or disease causing (MutationTaster), N: neutral (LRT) or polymorphism (MutationTaster), NA: missing data, PrD: probably damaging, PsD: possibly damaging, T: tolerated, U: Unknown.

## Abbreviations

CMTX5: X-linked Charcot-Marie-Tooth, type 5
DFN2: X-linked non-syndromic sensorineural deafness
Indel: Insertion/deletion polymorphism
OA: Optic atrophy
PRPP: Phosphoribosyl pyrophosphate
PRS: Phosphoribosyl pyrophosphate synthetase
RP: Retitinis pigmentosa
SNV: Nonsynonymous single nucleotide variant
WES: Whole exome sequencing
