# Supplementary material for: Expanding the phenotype of PRPS1 syndromes in females: neuropathy, hearing loss and retinopathy
Source: Orphanet J Rare Dis. 2014 Dec 10;9:190. doi: 10.1186/s13023-014-0190-9 (PMC4272780; doi:10.1186/s13023-014-0190-9)
Supplement: Additional file 1: — Clinical data of family RP-0482 and primers used in the study. [file 13023_2014_190_MOESM1_ESM.docx]

**Clinical evaluation of family RP-0482**

Diagnostic evaluations of patients were based on ophthalmological, neurological and otological examinations. Ophthalmological evaluation included the assessment of visual acuity, intraocular pressure, ocular motility, pupillary reaction, biomicroscopic slit-lamp examination, dilated fundus examination, best-corrected visual acuity, visual-field tests, fundoscopy, autofluoresce, optical coherence tomography examinations and full-field and multifocal electroretinogram (ERG) responses. Visual function was performed by static perimetry, D15 panel testing and Ganzfeld electroretinography according to the International Society for Clinical Electrophysiology of Vision Standards [1] with a UTAS 2000 system (LKC Technologies, Gaithersburg, USA) and jet electrodes.

Diagnosis of RP was based on clinical evaluation of night blindness, progressive visual field constriction, and poor visual acuity, and confirmed by altered or abolished ERG responses. Neurological evaluation included examination of cognitive status, cranial nerves, deep tendon reflexes, sensory and motor systems evaluations, and cerebellar testing. MRI was also performed and hearing assessment was done by pure tone audiometry and otoscopic examination.

**Clinical data**

**Proband (IV:3)**

*History:* Patient IV:3 suffered from congenital horizontal nystagmus in both eyes. Her parents reported her first walking at the age of two and having gait abnormalities since then. At the age of four, she started experiencing night blindness and loss of VA and she was first diagnosed with optic atrophy (OA) when she was five and with RP at 14. The patient was first studied in our hospital at 19 years of age. Ophthalmological examination at this age showed constriction of visual field to central 20º and loss of VA to 20/200 in both eyes. Both scotopic and photopic ERG responses were not recordable above noise and the visual evoked potentials (VEP) responses were delayed. Posterior subcapsular cataract was found in both eyes. Fundus showed pale and atrophic papilla, narrowed vessels, bone-like spicule pigmentations and retinal pigment epithelium (RPE) atrophy at the mid periphery of both eyes, and a well-defined macular lesion in the right eye. Neurological examination showed moderate psychomotor retardation, mild intellectual disability, loss of strength, *pes cavus* and bilateral and symmetrical cerebellar alteration. Biopsy of skeletal muscle showed the presence of striated muscle tissue with signs of re-innervation. Head MRI at 20 years did not show any significant alteration. Audiometry testing evidenced asymmetric sensorineural hypoacusis in both ears (90Dbs right ear and 30Dbs left ear) that currently has progressed to need hearing aid.

*Examination at 34 years*: Over the last 15 years, she has shown a slow progressive VA loss to hand motion in the right eye and to < 20/400 in the left eye. The macular atrophy has also progressed in both eyes as revealed by OCT, funduscopy and autofluorence images (Figure 2A-D). Her latest neurological evaluation evidenced symmetrical Charcot-Marie-Tooth-like sensorimotor polyneuropathy with cranial nerves unaltered, biceps and triceps reflex altered and radius, knee and ankle reflexes abolished. The motor evaluation showed muscle strength altered in distal lower limb and in proximal upper limbs. She also displayed vibratory, touch and algesic distal sensory loss, *pes cavus* and atrophy in both hands. Cerebellar examination evidenced dysmetria, ataxia and intention tremor and head MRI confirmed moderate cerebellar atrophy.

**Affected sister (IV:2)**

*History*: Patient IV:2 has no history of psychomotor or intellectual delay. She complained of slightly poor night vision at the age of 16 and OA was suspected due to altered responses on VEP. She was first studied in our hospital when she was 23 years of age, when an ophthalmological exam evidenced both eyes exhibiting pale optic disc, slightly attenuated retinal vessels and bone spicules at the extreme mid-periphery and conserved macula. She also showed some peripheral scotomes in the right eye, however, she had normal ERG responses and VA was not reduced (20/20). Auditory and neurological examinations were also normal.

*Examination at the age of 36:* The patient complained of photophobia and photopsias but showed no loss of peripheral vision and/or VA. Funduscopy evidenced only minor changes compared to the first exams, including a slight RPE atrophy. Full-field ERG exhibited normal scotopic and photopic responses but multifocal ERG revealed significantly lower responses at perifoveal regions with foveal preservation. OCT images confirmed an atrophy of the photoreceptors layer in the perifoveal region and the normal foveal thickness. Neurological evaluation was normal and head MRI did not show cerebellar atrophy.

**Affected mother (III:2)**

*History*: Patient III:2 complained of night blindness, loss of VA and constrained visual field since childhood but she was not diagnosed with RP until 47 years of age. At this age, she presented loss of VA (20/200, right eye and 20/25, left eye). The VEP responses were abolished in the right eye and very altered in the left. She was diagnosed with an asymmetric sensorineural hearing loss (40Dbs in left ear and normal in right ear) at 50 years. The patient was first studied in our hospital at 55 years of age. Ophthalmological examination at this age showed constriction of visual field and progressive loss of VA to counting fingers in the right eye and to 20/50 in the left eye. Scotopic ERG responses were not recordable and photopic responses were very diminished. Fundus showed pale and atrophic papilla, narrowed vessels and sparse spicule pigmentations in both eyes. Right eye also manifested patches of atrophy at the mid periphery and macular regions.

*Examination at 70 years:* The patient’s latest ophthalmological examination showed a VA of counting fingers in the RE and of 20/400 in the LE, and absence of scotopic and photopic ERG responses.. Funduscopy reflected a very pale and atrophic papilla, narrowed vessels, choriocapillar atrophy and sparse spicule pigmentations in both eyes. The right eye displayed a very well defined macular lesion of atrophy and the left presented an alteration in RPE. Neurological examination evidenced asymmetrical sensorimotor polyneuropathy, with slight loss of strength, and mild bilateral thenar muscle atrophy. The sensory evaluation demonstrated a deep sensory loss. Cerebellar exploration revealed mild intention tremor and ataxia (8.5 in the Scale for the Assessment and Rating of Ataxia -SARA-), a slight dysmetria, predominant in left upper and lower limbs, and *pes cavus*. Head MRI exhibited a mild cerebellar atrophy.

**Previous genetic screenings in the family**

Genomic DNA samples were extracted from peripheral blood lymphocytes from all participating individuals of the family and controls (BioRobot EZ1 Qiagen, Hilden, Germany). Additionally, DNA from saliva (Oragene containers, DNA Genotek), and total RNA from peripheral blood (PAXgene blood RNA kit, Qiagen) were extracted from individuals III:2, IV:1, IV:2 and IV:3.

Known mutations in autosomal dominant RP (adRP) genes were previously excluded in the index case using a genotyping microarray based on Arrayed Primer Extension (APEX) technology (adRP chip, Asper Ophthalmics, Tartu, Estonia). Differential diagnosis was performed with other pathologies with overlapping symptoms such as mithocondriopathies, ataxic forms of RP and Refsum disease. Mitochondrial mutations in *MTTH*, *MTTK*, *MTTS2*, and *MTTL2* and those associated with NARP (Neurogenic muscle weakness, Ataxia, and Retinitis Pigmentosa, MIM 551500) and Leigh Syndrome (MIM 256000) were discarded using Sanger sequencing. Screening for mutations in *SCA7*, associated with a form of ataxia with RP, spinocerebellar ataxia type 7 (MIM 164500), and peroxisomal function testing for Refsum disease (MIM 266500) were negative. Karyotype of the proband was also normal

**Primer sequences used in the study**

**Primers used for validation and segregation of the candidate mutations in family RP-0482**

| **Position** | **Gene** | **Aminoacid change** | **Reference Sequence** | **Sequence_Primer_F (5’>3’)** | **Sequence_Primer_R (5’>3’)** |
| --- | --- | --- | --- | --- | --- |
| chr7:48312363 | ABCA13 | p.L1034I | NM_152701 | GGCTCTTCGTTGACTTTCCTT | CCAGTTCTTGGCCCTCTGTA |
| chr16:3554786 | CLUAP1 | p.P30R | NM_015041 | TGTGATGGGGAACTGAATGA | ACTTTCCCTTTTGCAGCATC |
| chr13:99099055 | FARP1 | p.E1014K | NM_005766 | GCTCACCATCCCCTCTGAGT | ACTCGCTGACTTGGGAGAAA |
| chr8:11687784 | FDFT1 | p.D78G | NM_004462 | CTATGCACACGCTGACCTGT | CCCTTCCCTACCTGTGGAAT |
| chr1:203033044 | PPFIA4 | p.M24T | NM_015053 | CTTCCTCCCCCATAATAGCC | GAGGCAGACCCTCAGAGCTA |
| chrX:106871904 | PRPS1 | p.S16P | NM_002764 | ATTGAGTCTGTGGCCGACTT | GGGCAGGTGAGGTCTAGTCA |
| chr3:51696477 | RAD54L2 | p.G475C | NM_015106 | TTTGGGGACTACAGGACAGG | ATGATCTCTGGGCTGTCAGG |
| chr1:110883886 | RBM15 | p.G620A | NM_022768 | TCGGACTGCAGCTACTTCTG | ATCCAGATGACGTCCTCCAC |
| chr3:50112732 | RBM6 | p.Y414C | NM_005777 | GATGGTGTCTGCTTCTTTTGC | GGCAAAGACATCACTCTAGGC |
| chr1:84963141 | RPF1 | p.K346N | NM_025065 | AACCCGGTTTTTCAGCACTA | ATTCGTCCAGACGTGAAAGG |
| chr18:74617295 | ZNF236 | p.R739C | NM_007345 | GGAAGAGGCTTTGTTTCTGC | CACCAGAATTACACCCACCTG |
| chr2:135975092 | ZRANB3 | p.P811R | NM_032143 | TCTGCCTGTGAGCTGTTAACTC | CCCCTGTAGAAAGAACCATGA |

**Primers used for *PRPS1* mRNA expression assays and cDNA sequencing**

Exon 1-2:

Forward: 5’-CCGTGATCGCTTAGTGGAGT-3’

Reverse: 5’-GATTTCGCCACAACCACTCT-3’

Exons 1-4:

Forward: 5’-CCGTGATCGCTTAGTGGAGT-3’

Reverse: 5’-ACCAGCATCAGGTGAGACAA-3’

Exons 4-7:

Forward: 5’-AGAGCCGGCTGTCCTAAAGT-3’

Reverse: 5’-GGGTGGGGTGGATTTTATTC-3’

**Primers used for X-chromosome inactivation**

Primers used to detect the methylated allele:

Forward: 5’-GCGAGCGTAGTATTTTTCGGC-3’

Reverse: 5’-AACCAAATAACCTATAAAACCTCTACG-3’

Primers used to detect the unmethylated allele:

Forward: 5’-GTTGTGAGTGTAGTATTTTTTGGT-3’

Reverse: 5’-CAAATAACCTATAAAACCTCTACA-3’

**References**

1. Marmor MF, Zrenner E. **Standard for clinical electro-oculography. International Society for Clinical Electrophysiology of Vision**. *Arch Ophthalmol* 1993;**111**:601-4.
