# Supplementary figures and images for: Expanding the phenotype of PRPS1 syndromes in females: neuropathy, hearing loss and retinopathy
Source: Orphanet J Rare Dis. 2014 Dec 10;9:190. doi: 10.1186/s13023-014-0190-9 (PMC4272780; doi:10.1186/s13023-014-0190-9)

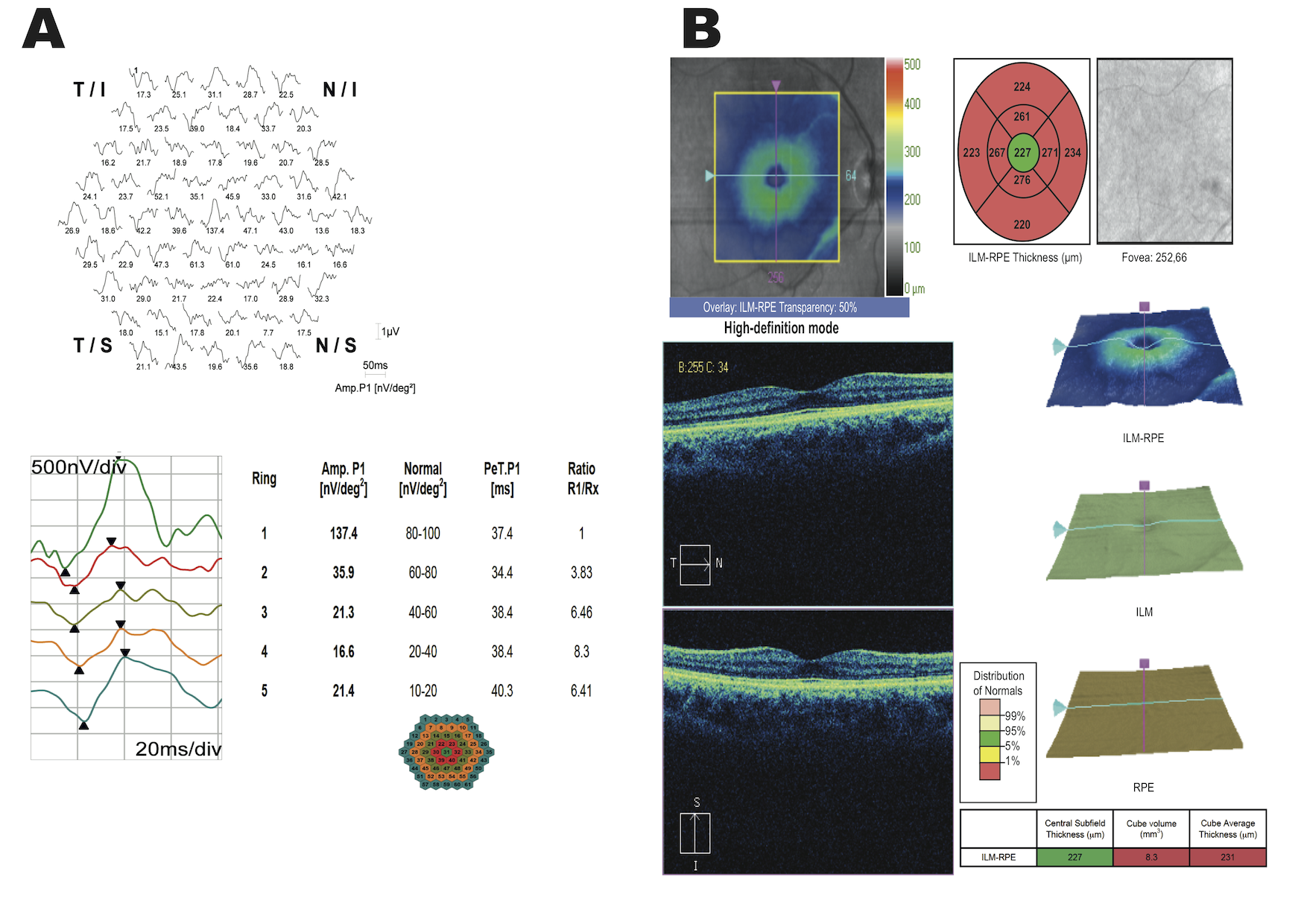

Supplement: Additional file 2: Figure S1. — Multifocal ERG and Optical coherence tomography (OCT) findings suggested a sectorial affectation of perifoveal photoreceptors. A. Right eye multifocal ERG (mfERG) records in patient IV:2 demonstrate a reduced function of photoreceptors of perifoveal region with preserved function of fovea. The ring analysis (see schematic) goes from the centre to the periphery. Quantitative results of mfERG analyses are displayed in table format. B. OCT macular cube 512×128 scan show macular thickness in the right eye from patient IV:2. ILM: inner limiting membrane; RPE: retinal pigment epithelium. Top left: fundus image with scan cube overlay. Top right: macular thickness significance map. The central innermost 1-mm-diameter circle represents the central subfield; inner superior, inner nasal, inner inferior, and inner temporal areas bounded by the 3-mm-diameter circle form the inner macula; outer superior, outer nasal, outer inferior, and outer temporal areas bounded by the 6-mm-diameter circle form the outer macula. Retinal thickness values from ILM to RPE are compared to the normative data. Middle and bottom left: cross-sectional OCT scans. Middle right: 3days surface maps: the ILM-RPE, displaying the retinal thickness in three dimensions. Bottom right: central subfield thickness, overall average macular thickness, and overall macular volume compared to normative data are displayed in table format. Reduction in the retinal thickness in the perifoveal region with a normal foveal thickness is also evidenced. [file 13023_2014_190_MOESM2_ESM.tiff]
