# Supplementary material for: Expanding the phenotype of PRPS1 syndromes in females: neuropathy, hearing loss and retinopathy
Source: Orphanet J Rare Dis. 2014 Dec 10;9:190. doi: 10.1186/s13023-014-0190-9 (PMC4272780; doi:10.1186/s13023-014-0190-9)
Supplement: Additional file 3: Table S1 and S2. — Genetic variants obtained (single nucleotide variants –SNV-, Table S1, and insertion deletion variants –indels-, Table S2) after the first variant filtering analysis. Table S3. Variants yielded by the second filtering of the 141 variants based on the novelty, conservation across species according to the values from PhyloP and GERP++, predicted pathogenicity at least by two of the systems evaluated and expression in the retina. 1KGP: Minor allele frequency in the 1000 Genome Project, ESP: Minor allele frequency in the NHLBI GO Exome Sequencing Project. For PhyloP prediction, C: Conserved, NC: Not conserved. For pathogenicity prediction systems, B: benign, D: deleterious (LRT), or damaging (SIFT) or disease causing (MutationTaster), N: neutral (LRT) or polymorphism (MutationTaster), NA: missing data, PrD: probably damaging, PsD: possibly damaging, T: tolerated, U: Unknown. [file 13023_2014_190_MOESM3_ESM.docx]

**Supplemental Tables**

**Supplemental tables S1 and S2:** Genetic variants obtained (single nucleotide variants –SNV-, table 1, and insertion deletion variants –indels-, table 2) after the first variant filtering analysis.

**Supplemental table S3:** Variants yielded by the second filtering of the 141 variants based on the novelty, conservation across species according to the values from PhyloP and GERP++, predicted pathogenicity at least by two of the systems evaluated and expression in the retina

1KGP: Minor allele frequency in the 1000 Genome Project, ESP: Minor allele frequency in the NHLBI GO Exome Sequencing Project. For PhyloP prediction, C: Conserved, NC: Not conserved. For pathogenicity prediction systems, B: benign, D: deleterious (LRT), or damaging (SIFT) or disease causing (MutationTaster), N: neutral (LRT) or polymorphism (MutationTaster), NA: missing data, PrD: probably damaging, PsD: possibly damaging, T: tolerated, U: Unknown.

**Table S1**

| **Chr** | **Position** | **rs id** | **1KGP** | **ESP** | **Aminoacid change** | **Gene** | **GERP++** | **PhyloP** | **LRT** | **Poly**  **Phen2** | **SIFT** | **MutationTaster** |
| --- | --- | --- | --- | --- | --- | --- | --- | --- | --- | --- | --- | --- |
| **1** | 12089299 |  |  |  | p.R226C | *MIIP* | 2.33 | C | N | PrD | T | N |
| **1** | 27240176 |  |  |  | p.R86W | *NR0B2* | 2.78 | C | N | PsD | D | N |
| **1** | 52954676 |  |  |  | p.D474N | *ZCCHC11* | 5.04 | C | D | PrD | T | N |
| **1** | 84963141 |  |  |  | p.K346N | *RPF1* | 5.61 | C | D | PsD | D | D |
| **1** | 110883886 |  |  |  | p.G620A | *RBM15* | 4.42 | C | D | B | T | D |
| **1** | 117663358 | rs139632476 | 0.0005 | 0.0008 | p.H156Y | *TRIM45* | 4.65 | C | N | PrD | T | N |
| **1** | 151739750 |  |  |  | p.H48R | *OAZ3* |  |  |  |  |  |  |
| **1** | 152659479 | rs148459371 | 0.0005 | 0.0005 | p.G54S | *LCE2B* | -0.91 | NC | NA | NA | D | N |
| **1** | 154316895 |  | 0.0005 |  | p.S720F | *ATP8B2* | 5.3 | C | D | PrD | D | D |
| **1** | 155221575 |  |  | 0.0001 | p.P14L | *FAM189B* | 4.48 | C | D | PrD | D | N |
| **1** | 155233070 |  |  |  | p.A252V | *CLK2* | 4.97 | C | N | B | T | N |
| **1** | 156756783 |  |  |  | p.E300D | *PRCC* | 1.97 | NC | N | B | T | D |
| **1** | 158262505 |  |  |  | p.Q244K | *CD1C* | -0.353 | NC | N | B | T | N |
| **1** | 172571942 |  |  | 0.0001 | p.I1094L | *C1orf9* |  |  |  |  |  |  |
| **1** | 186092095 |  |  | 0.0001 | p.I4081T | *HMCN1* | 5.52 | C | D | PrD | D | D |
| **1** | 203033044 |  |  |  | p.M24T | *PPFIA4* |  |  |  |  |  |  |
| **1** | 219366546 | rs145589411 | 0.0005 | 0.0012 | p.I105T | *LYPLAL1* | 5.48 | C | D | PrD | D | D |
| **1** | 229730691 | rs41304137 | 0.0009 | 0.0018 | p.T375A | *TAF5L* | 4.6 | C | D | PsD | D | D |
| **1** | 245861438 |  |  | 0.0001 | p.L1952P | *KIF26B* |  |  |  |  |  |  |
| **1** | 247614909 |  |  |  | p.R126C | *OR2B11* | 3.3 | C | N | PrD | D | N |
| **2** | 75882376 | rs189220165 | 0.0005 |  | p.I282V | *MRPL19* | 2.21 | NC | D | B | T | N |
| **2** | 133542717 |  |  | 0.0003 | p.T556M | *NCKAP5* |  |  |  |  |  |  |
| **2** | 135621175 |  |  |  | p.A154T | *ACMSD* | 1.34 | C | D | B | T | D |
| **2** | 135975092 |  |  |  | p.P811R | *ZRANB3* |  |  |  |  |  |  |
| **2** | 160697271 | rs147904044 | 0.0023 | 0.0041 | p.S1159F | *LY75,LY75-CD302* | 3.11 | C | NA | PrD | T | NA |
| **2** | 160735174 | rs147820690 | 0.0014 | 0.0022 | p.G525E | *LY75,LY75-CD302* | 4.37 | C | N | PrD | D | NA |
| **2** | 171687495 |  |  | 0.0001 | p.V114M | *GAD1* | 5.55 | C | D | B | T | N |
| **2** | 217001820 | rs192418356 | 0.0005 |  | p.V261F | *XRCC5* | 3.95 | NC | D | PrD | D | D |
| **3** | 49940430 |  |  | 0.0001 | p.A205T | *MST1R* | -8.91 | NC | N | B | T | N |
| **3** | 50112732 |  |  |  | p.Y414C | *RBM6* | 3.34 | C | N | PrD | D | D |
| **3** | 51696477 |  |  |  | p.G475C | *RAD54L2* | 4.93 | C | D | PrD | D | D |
| **3** | 75714929 | rs148715408 |  |  | p.A196T | *FRG2C* | -1.33 | NC | N | PsD | T | N |
| **3** | 75714950 | rs147607693 |  |  | p.L203M | *FRG2C* | 0.8 | NC | N | B | T | N |
| **3** | 120352045 |  |  |  | p.E238D | *HGD* | 0.531 | NC | D | B | T | D |
| **3** | 167245750 | rs147197491 | 0.0005 | 0.0002 | p.R294I | *WDR49* | 3.4 | NC | N | PrD | T | N |
| **3** | 195452987 | rs9866681 |  |  | p.A299T | *MUC20* |  |  |  |  |  |  |
| **3** | 195452991 | rs3828411 |  |  | p.T300I | *MUC20* |  |  |  |  |  |  |
| **4** | 48993551 |  |  |  | p.V106L | *CWH43* | 2.72 | C | N | B | T | D |
| **4** | 106158350 | rs75056899 | 0.0027 | 0.0026 | p.Q1084P | *TET2* | 0.37 | C | N | PsD | D | N |
| **4** | 147755012 | rs41280533 |  | 0.0014 | p.D308V | *TTC29* |  |  |  |  |  |  |
| **4** | 148559898 | rs76148346 | 0 | 0.0008 | p.V419I | *PRMT10:PRMT10* | 4.85 | C | D | PsD | D | N |
| **5** | 149301253 | rs114973968 | 0.0037 | 0.0036 | p.P212L | *PDE6A* | 5.06 | C | D | PrD | D | D |
| **5** | 176008381 |  |  |  | p.R619H | *CDHR2* | -5.02 | NC | NA | PrD | NA | N |
| **5** | 179192490 | rs141968004 |  | 0.0001 | p.P160H | *MAML1* | 4.59 | C | D | PrD | T | D |
| **6** | 31238909 | rs1050686 |  |  | p.T66K | *HLA-B,HLA-C* | -4.24 | NC | U | B | T | NA |
| **6** | 31238910 | rs1050685 |  |  | p.T66A | *HLA-B,HLA-C* | -4.24 | NC | U | B | T | NA |
| **6** | 56483679 | rs45487998 | 0.0037 | 0.0023 | p.K1718T | *DST* | 2.75 | NC | NA | NA | T | N |
| **6** | 74528127 | rs35238647 | 0.0018 | 0.0042 | p.R1233G | *CD109* | 2.47 | NC | U | PsD | T | N |
| **7** | 48312363 |  |  |  | p.L1034I | *ABCA13* |  |  |  |  |  |  |
| **7** | 99084275 | rs138157337 | 0.0005 | 0.0009 | p.D90N | *ZNF789* | -1.74 | NC | NA | B | T | N |
| **7** | 131895706 |  | 0.0014 | 0.0002 | p.T765I | *PLXNA4* | 4.86 | C | N | PsD | NA | D |
| **7** | 138269570 | rs34585297 | 0.0009 | 0.0023 | p.R975S | *TRIM24* | 3.2 | C | N | B | T | N |
| **8** | 11687784 |  |  |  | p.D78G | *FDFT1* | 5.57 | C | D | PrD | D | D |
| **8** | 37691254 |  |  |  | p.V449M | *GPR124* | 3.06 | C | D | B | T | N |
| **8** | 56723438 |  |  |  |  | *TGS1* |  |  |  |  |  |  |
| **8** | 98041687 |  |  |  | p.G340R | *PGCP* | 5.29 | C | D | B | T | D |
| **8** | 146156974 |  |  |  | p.T400N | *ZNF16* | 3.55 | C | NA | B | D | N |
| **9** | 35704339 | rs144809355 | 0.0009 | 0.0006 | p.A2013T | *TLN1* | 4.65 | C | D | PsD | D | D |
| **9** | 36249368 |  |  |  | p.K26N | *GNE* |  |  |  |  |  |  |
| **9** | 37304236 | rs138176259 | 0.0014 | 0.0024 | p.S236A | *ZCCHC7* | -2.19 | NC | N | B | T | N |
| **9** | 78808173 | rs148131474 |  | 0.0008 | p.E157K | *PCSK5* | 5.2 | C | NA | PrD | D | N |
| **10** | 7763669 | rs147906523 |  | 0.0004 | p.E266Q | *ITIH2* | 3.86 | C | N | B | T | D |
| **10** | 46999289 | rs140859626 | 0.0027 | 0.0018 | p.R137W | *GPRIN2* | 1.69 | C | N | PrD | D | N |
| **10** | 98819233 |  |  |  | p.R357C | *SLIT1* | 4.44 | C | D | PrD | D | D |
| **10** | 102781975 |  |  |  | p.Y237C | *PDZD7* | 4.94 | C | D | PrD | D | D |
| **10** | 105793895 | rs150282756 | 0.0018 | 0.0004 | p.G1322S | *COL17A1* | 4.1 | C | N | NA | D | D |
| **10** | 124914494 |  |  |  | p.K21E | *BUB3* | 2.98 | NC | D | B | D | D |
| **10** | 134646912 |  |  |  | p.R2356Q | *TTC40* | 3.49 | C | D | NA | NA | N |
| **11** | 5701205 |  |  |  | p.R68Q | *TRIM5* | 2.78 | C | N | B | T | N |
| **11** | 126126712 | rs180953673 | 0.0009 |  | p.R316Q | *FAM118B* | 4.67 | C | D | PrD | T | D |
| **12** | 10954919 | rs144677090 |  | 0.0003 | p.I84T | *TAS2R7* | 4.01 | C | N | PsD | T | N |
| **12** | 10978391 |  |  |  | p.T160A | *TAS2R10* | -4.78 | NC | N | B | T | N |
| **12** | 118517228 |  | 0.0014 | 0.0012 | p.S283L | *VSIG10* |  |  |  |  |  |  |
| **12** | 133277845 |  |  |  | p.A137T | *PXMP2* | -10.3 | NC | N | B | T | NA |
| **13** | 39454452 | rs114400765 | 0 | 0.0019 | p.T3013M | *FREM2* | 5.42 | C | D | PrD | D | D |
| **13** | 99099055 |  |  |  | p.E1014K | *FARP1* | 5.1 | C | D | PrD | T | D |
| **14** | 23745014 |  | 0.0018 | 0.002 | p.Q475E | *HOMEZ* |  |  |  |  |  |  |
| **14** | 80997207 |  |  | 0.0002 | p.H968Q | *CEP128* | 5.57 | C | D | PrD | T | N |
| **14** | 93649990 | rs35855685 |  | 0.0037 | p.V200I | *MOAP1* | 2.83 | C | NA | B | T | N |
| **14** | 107034967 | rs72686844 |  |  | p.K38R | *immunoglobulin heavy chain* |  |  |  |  |  |  |
| **15** | 22867569 |  |  |  | p.R882H | *TUBGCP5* | 3.9 | C | D | PrD | D | D |
| **15** | 40915719 |  |  |  | p.M936T | *CASC5* | 0.922 | NC | N | PrD | T | N |
| **15** | 76914159 | rs184003295 | 0.0023 | 0.0038 | p.Y194F | *SCAPER* |  |  |  |  |  |  |
| **15** | 86236607 |  |  |  | p.I42V | *AKAP13* | 1.73 | NC | NA | B | T | N |
| **15** | 91545322 |  |  |  | p.V364L | *VPS33B* | 2.89 | NC | D | PsD | T | D |
| **16** | 1400112 | rs142109530 | 0.0041 | 0.0022 | p.A217V | *C16orf42* | 0.819 | NC | N | B | T | N |
| **16** | 2546790 |  | 0 | 0.0011 | p.R214H | *TBC1D24* | 5.08 | C | D | PrD | NA | D |
| **16** | 2979735 |  |  |  | p.G17S | *FLYWCH1* |  |  |  |  |  |  |
| **16** | 3554786 |  |  |  | p.P30R | *CLUAP1* | 4.02 | C | D | PrD | D | D |
| **16** | 3640008 |  | 0.0005 |  | p.E1211K | *SLX4* | 3.46 | NC | N | B | D | N |
| **16** | 3640271 | rs144647122 |  | 0.0008 | p.S1123Y | *SLX4* | 5.72 | C | N | PsD | T | N |
| **16** | 4835833 |  |  | 0.0001 | p.G117R | *SEPT12* | 4.17 | C | D | PrD | D | D |
| **16** | 71103279 |  |  |  | p.Y367C | *HYDIN* | -2.82 | NC | U | PsD | T | N |
| **16** | 88705476 | rs189998965 | 0.0027 | 0.0041 | p.G32S | *IL17C* | -1.27 | NC | N | PsD | NA | N |
| **17** | 7758480 |  |  |  | p.L30V | *TMEM88* | 4.29 | C | D | PsD | D | D |
| **17** | 21319868 | rs73979902 |  |  | p.S405I | *KCNJ12,KCNJ18* | 5.16 | C | N | B | D | D |
| **17** | 26905104 | rs138772502 | 0.0023 | 0.0017 | p.N1145T | *SPAG5* | 4.28 | C | N | PrD | T | N |
| **17** | 33591411 |  |  |  | p.P450A | *SLFN5* | 3.02 | C | N | PrD | T | N |
| **17** | 73567097 | rs74876688 | 0.0018 | 0.0008 | p.R325C | *LLGL2* | 3.07 | C | D | PrD | D | NA |
| **17** | 73760020 |  |  |  | p.R105W | *GALK1* | 4.75 | C | N | PsD | D | N |
| **17** | 76547636 | rs61740096 | 0.0046 | 0.0049 | p.G493V | *DNAH17* |  |  |  |  |  |  |
| **17** | 76795061 |  |  |  | p.I117V | *USP36* | 1.37 | NC | N | PsD | D | N |
| **17** | 78318686 | rs138595111 |  | 0.0022 | p.Q2184R | *RNF213* | -1.22 | NC | N | B | T | N |
| **17** | 80443515 |  |  |  | p.L324F | *NARF* | 2.47 | NC | D | PsD | D | D |
| **18** | 74617295 |  |  |  | p.R739C | *ZNF236* | 5.34 | C | D | PrD | D | D |
| **19** | 10288010 |  |  | 0.0002 | p.K160R | *DNMT1* | 2.86 | C | N | B | T | N |
| **19** | 10671694 | rs142099787 | 0.0018 | 0.0012 | p.D218E | *KRI1* | -4.51 | NC | N | B | T | N |
| **19** | 14938860 | rs61731995 | 0.0014 | 0.0015 | p.N65S | *OR7A5* | 0.346 | NC | U | PrD | T | N |
| **20** | 30371542 |  |  |  | p.I411L | *TPX2* | 5.35 | C | D | PrD | T | NA |
| **21** | 14982886 | rs142207431 |  |  | p.G113S | *POTED* | 0 | NC | NA | B | T | N |
| **21** | 16338443 | rs61755058 | 0.0023 | 0.0022 | p.P691S | *NRIP1* | -2.95 | NC | N | B | D | D |
| **21** | 46595744 |  |  |  | p.N92S | *ADARB1* | 2.58 | NC | N | PrD | T | D |
| **22** | 24579049 | rs35660748 |  |  | p.R34H | *SUSD2* | 3.13 | C | N | PsD | D | N |
| **22** | 39411686 |  |  |  | p.L35P | *APOBEC3C* | 0.886 | NC | NA | PrD | D | N |
| **22** | 39777839 |  |  |  | p.A208T | *SYNGR1* | -4.48 | NC | N | NA | T | N |
| **X** | 35971793 |  | 0.0006 | 0.0003 | p.S377R | *CXorf22* | 2.51 | NC | D | PrD | D | N |
| **X** | 38664407 |  |  |  | p.V70I | *MID1IP1* | 1.74 | NC | N | B | T | N |
| **X** | 69668802 | rs140249987 |  | 0.0005 | p.V133I | *DLG3* | 4.18 | C | D | B | T | N |
| **X** | 106871904 |  |  |  | p.S16P | *PRPS1* | 4.1 | C | D | B | D | D |

**Table S2**

| **Chr** | **Position** | **rs id** | **Reference allele** | **Alternate allele** | **AA change** | **Effect** | **Gene** | **Impact** |
| --- | --- | --- | --- | --- | --- | --- | --- | --- |
| **1** | 14106394 | rs148293494 | A | ACTC | T501TP | Codon insertion | *PRDM2* | Moderate |
| **1** | 109465165 | rs111974813 | ACTT | A | TS523T | Codon deletion | *GPSM2* | Moderate |
| **1** | 152681680 | rs11269814 | C | CAGCTCTGGGGGCTGCTGT | -44SSGGCC | Codon insertion | *LCE4A* | Moderate |
| **2** | 38179414 | rs141540819 | CTG | C |  | Frame shift | *FAM82A1* | High |
| **3** | 97983487 | rs11279406 | TTGTAACCAC | T | LVTT120L | Codon deletion | *OR5H6* | Moderate |
| **5** | 156479443 | rs143959546 | CTTG | C | TS200S | Codon change plus deletion | *HAVCR1* | Moderate |
| **6** | 49437853 | rs11333073 | AT | A |  | Splice site acceptor | *CENPQ* | High |
| **6** | 82461727 |  | ACCGCCGAAGTCGCCG | A | GGDFGG120G | Codon change plus deletion | *FAM46A* | Moderate |
| **9** | 107360768 | rs11314210 | GT | G |  | Frame shift | *OR13C5* | High |
| **9** | 107367392 | rs143198170 | TGTTA | T |  | Frame shift | *OR13C2* | High |
| **9** | 107367664 | rs143760725 | AGC | A |  | Frame shift | *OR13C2* | High |
| **11** | 4790873 | rs141621954 | CG | C |  | Frame shift | *OR51F1* | High |
| **11** | 48285981 | rs112100800 | CCTT | C | TF190T | Codon deletion | *OR4X1* | Moderate |
| **17** | 38858134 | rs11309872 | CA | C |  | Frame shift | *KRT24* | High |
| **17** | 39240781 |  | CCTGCTGCCGCCCCAG | C |  | Frame shift | *KRTAP4-9* | High |
| **19** | 49657710 | rs10533934 | ACAT | A | DV231V | Codon change plus deletion | *HRC* | Moderate |
| **20** | 44520237 | rs10582052 | CCTG | C | L11- | Codon deletion | *CTSA* | Moderate |
| **21** | 11029596 | rs138714104 | AC | A |  | Splice site donor | *TPTE* | High |

**Table S3**

| **Position** | **Aminoacid change** | **Gene** | **GERP++** | **PhyloP** | **LRT** | **PolyPhen2** | **SIFT** | **MutationTaster** |
| --- | --- | --- | --- | --- | --- | --- | --- | --- |
| chr7:48312363 | p.L1034I | *ABCA13* |  | Conserved (UCSC) |  |  |  |  |
| chr16:3554786 | p.P30R | *CLUAP1* | 4.02 | C | D | PrD | D | D |
| chr13:99099055 | p.E1014K | *FARP1* | 5.1 | C | D | PrD | T | D |
| chr8:11687784 | p.D78G | *FDFT1* | 5.57 | C | D | PrD | D | D |
| chr1:203033044 | p.M24T | *PPFIA4* |  | Conserved (UCSC) |  |  |  |  |
| chrX:106871904 | pS16P | *PRPS1* | 4.1 | C | D | B | D | D |
| chr3:51696477 | p.G475C | *RAD54L2* | 4.93 | C | D | PrD | D | D |
| chr1:110883886 | p.G620A | *RBM15* | 4.42 | C | D | B | T | D |
| chr3:50112732 | p.Y414C | *RBM6* | 3.34 | C | N | PrD | D | D |
| chr1:84963141 | p.K346N | *RPF1* | 5.61 | C | D | PsD | D | D |
| chr18:74617295 | p.R739C | *ZNF236* | 5.34 | C | D | PrD | D | D |
| chr2:135975092 | p.P811R | *ZRANB3* |  | Conserved (UCSC) |  |  |  |  |
